# Supplementary material for: Prioritizing COVID-19 vaccine allocation in resource poor settings: Towards an Artificial Intelligence-enabled and Geospatial-assisted decision support framework
Source: PLoS One. 2023 Aug 10;18(8):e0275037. doi: 10.1371/journal.pone.0275037 (PMC10414619; doi:10.1371/journal.pone.0275037)
Supplement: S1 Table — (DOCX) [file pone.0275037.s001.docx]

**S1 Table.** **Vulnerability Factors.** These factors are included in the Social Vulnerability Index calculations from [1].

| **ID** | **Grp.** | **Factors** | **Definition** |
| --- | --- | --- | --- |
| 1 | **Socioeconomic** | Laborers | Percent of the population of adults between 15-49 that works in manual labour (construction, factories, drivers.) |
| 2 |  | Detergent/soap availability | Households’ percentage where use of soap or detergent was noticed |
| 3 |  | Car ownership | Households’ percentage that did not own a car |
| 4 |  | Basic hand washing facilities at home | Household percentage without a place for hand sanitisation or handwashing |
| 5 |  | Education attainment | Mean of years of schooling/education in area |
| 6 |  | Clean drinking water | Proportion of the household without access to improve source of clean water |
| 7 |  | Malnutrition | Prevalence of stunting |
| 8 |  | Households under poverty | Proportion of household with income within the lowest wealth quintile |
| 9 |  | Households sharing sanitation facilities | Percentage of household sharing |
| 10 | **Demographic** | Informal housing | Percentage of people living in informal housing or internally displaced people cams |
| 11 |  | Population aged 65+ | Percentage of population age 65 years old or more |
| 12 |  | Single parent families | Percentage of the population that are single parenting |
| 13 |  | Household crowding | Percentage of population living in shared bedroom with more than 3 people |
| 14 |  | Total population in log scale | Log of the total population in a unit area |
| 15 |  | Urban population | Percentage of the population living in urban areas |
| 16 | **Healthcare Accessibility** | Access to hospitals | Proportion of the population in 2 hours travel distance to healthcare facility |
| 17 |  | Health workforce | Ratio of clinical personnel per population |
| 18 |  | Hospital beds | Number of hospitals beds per population |
| 19 |  | Closeness to urban area | Travel time to the nearest urban area with more than 5,000 civilians |
