# Supplementary material for: Prioritizing COVID-19 vaccine allocation in resource poor settings: Towards an Artificial Intelligence-enabled and Geospatial-assisted decision support framework
Source: PLoS One. 2023 Aug 10;18(8):e0275037. doi: 10.1371/journal.pone.0275037 (PMC10414619; doi:10.1371/journal.pone.0275037)
Supplement: S1 Appendix — (DOCX) [file pone.0275037.s002.docx]

# S1 Appendix. Vulnerability Data

For the analysis presented in this paper we used data from the previous study on the COVID-19 vulnerability index in Kenya [1]. The list of factors used for the calculation of Social Vulnerability index and other vulnerability indexes are presented in S1 Table below.

However, there are more recent data that could be used for future analysis of the social vulnerability index:

- 2020 Kenya Malaria Indicator Survey [2],
- 2015-2016 Integrated Household Budget Survey (IHBS) [3],
- 2020 Kenya COVID-19 Rapid Response Phone Survey [4], and
- 1990-2015 gridded economic data [5].

Geospatial estimates of age and gender distributions can be retrieved from the WorldPop database which has been updated for 2020 for a broad array of countries [6].

In the case of Kenya, this gridded data can be aggregated at the sub-county level (presented in this paper) and at the ward level (third administrative layer) using a zonal statistics algorithm.

Finally, the share of population in each division which is living within 2 hours travel time from the nearest healthcare facility can be calculated and uploaded in the system [7]. This calculation considers surface friction represented by land barriers and transport infrastructure and the location of healthcare facilities is drawn from publicly available databases [8].

## References

[1] Macharia, P. M., Joseph, N. K., & Okiro, E. A. (2020). A vulnerability index for COVID-19: spatial analysis at the subnational level in Kenya. *BMJ global health*, *5*(8), e003014.

[2] Kenya: Demographic and Health Survey 2020. https://dhsprogram.com/methodology/survey/survey-display-579.cfm (accessed November 2021).

[3] Kenya National Bureau of Statistics (KNBS) (2015). Basic Report Kenya Integrated Household Budget Survey (KIHBS) 2015-2016. https://sun-connect-news.org/fileadmin/DATEIEN/Dateien/New/KNBS_-_Basic_Report.pdf (accessed June 2021).

[4] Delius, A., Himelein, K., & Pape, U. J. (2020). Conducting Rapid Response Phone Surveys to Fill Data Gaps.

[5] Kummu, M., Taka, M., & Guillaume, J. H. (2018). Gridded global datasets for gross domestic product and Human Development Index over 1990–2015. *Scientific data*, *5*(1), 1-15.

[6] Tatem, A. J. (2017). WorldPop, open data for spatial demography. *Scientific data*, *4*(1), 1-4.

[7] Falchetta, G., Hammad, A. T., & Shayegh, S. (2020). Planning universal accessibility to public health care in sub-Saharan Africa. *Proceedings of the National Academy of Sciences*, *117*(50), 31760-31769.

[8] South, A., Dicko, A., Herringer, M., Macharia, P. M., Maina, J., Okiro, E. A., ... & van der Walt, A. (2020). A reproducible picture of open access health facility data in Africa and R tools to support improvement. *Wellcome Open Research*, *5*.
